# Artificial Intelligence in Physical Therapy Education: Evaluating Clinical Reasoning Performance in Musculoskeletal Care Using ChatGPT

**DOI:** 10.1002/msc.70177

**Published:** 2025-07-31

**Authors:** Jie Hao, Zixuan Yao, Ka‐Chun Siu

**Affiliations:** ^1^ Department of Physical Therapy and Rehabilitation Southeast Colorado Hospital Springfield Colorado USA; ^2^ Department of Rehabilitation Medicine Beijing Hospital National Center of Gerontology Institution of Geriatric Medicine Chinese Academy of Medical Science Beijing P.R.China; ^3^ Department of Health & Rehabilitation Sciences College of Allied Health Professions University of Nebraska Medical Center Omaha Nebraska USA

## 1 Introduction

The integration of artificial intelligence (AI) in healthcare and medical education has expanded rapidly in recent years, with large language models such as ChatGPT emerging as accessible and versatile tools. Trained on enormous datasets, ChatGPT can generate human‐like responses to open‐ended prompts and has shown promise in tasks such as patient education, clinical documentation, and decision‐making support (Dave et al. 2023). Recent evaluations of ChatGPT have shown that it achieved 86% accuracy on the United States Medical Licensing Exam Step 1‐stye questions (Garabet et al. 2024), underscoring its potential to deliver consistent performance across a broad range of topics in medical education. In the context of physical therapy, ChatGPT demonstrated 80% concordance with clinical practice guideline recommendations for musculoskeletal care (Hao et al. 2025), suggesting its potential utility in supporting evidence‐based practice.

Clinical reasoning is a core competency in physical therapy education and clinical practice (Huhn et al. 2019). It encompasses the cognitive processes involved in gathering and interpreting patient information, generating hypotheses, conducting targeted examinations, synthesising findings, and formulating individualised management plans. Proficiency in clinical reasoning is essential for safe, effective, and evidence‐based care, particularly in the management of musculoskeletal conditions, which represent a significant portion of physical therapy practice. While previous studies have explored ChatGPT's application in medical education and various clinical tasks, its capacity to emulate the stepwise clinical reasoning process involved in a physical therapy patient encounter has not yet been examined.

This study seeks to address this gap by evaluating ChatGPT's clinical reasoning performance on musculoskeletal conditions in physical therapy. Understanding the strengths and limitations of ChatGPT in this context may inform its use in physical therapy education and help guide the development of AI‐augmented learning experiences that foster critical thinking and clinical competence.

## 2 Methods

**FIGURE 1:**
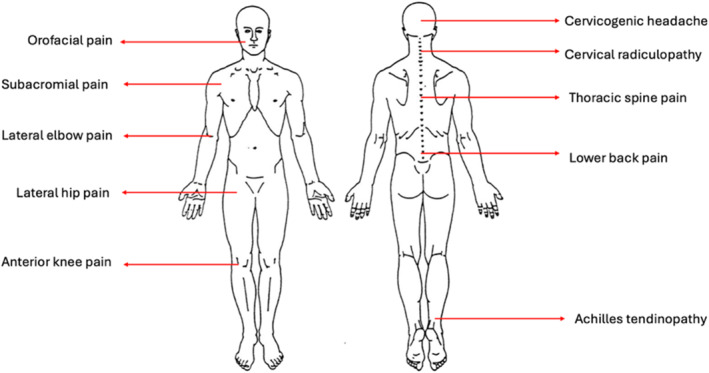
The 10 musculoskeletal case scenarios were used to evaluate ChatGPT's clinical reasoning performance.

This cross‐sectional study evaluated the clinical reasoning performance of ChatGPT‐4 across a series of simulated musculoskeletal physical therapy cases using a standardized framework based on the Subjective, Objective, Assessment, and Plan (SOAP) model (Delitto and Snyder‐Mackler 1995). Ten case scenarios were developed by two physical therapists (J.H. and Z.Y.) and included diverse musculoskeletal conditions: orofacial pain, shoulder pain, lateral elbow pain, lateral hip pain, knee pain, headache, neck pain, thoracic spine pain, low back pain, and Achilles' tendinopathy (Figure 1). Together, they encompass upper and lower extremity conditions, spinal disorders, and craniofacial dysfunction, providing a representative sample of the body regions routinely addressed in musculoskeletal physical therapy. Each case included patient history and a staged clinical examination (subjective and objective), simulating the typical flow of a physical therapy evaluation.

ChatGPT was first prompted with the following instructions:

“You are a physical therapist evaluating a patient with a musculoskeletal condition. I will provide you with clinical information in stages, following the typical flow of a physical therapy examination (subjective, objective, assessment, and plan). At each stage, I will ask you one clinical reasoning question related to that component of the SOAP framework. Please answer each question based on the information available at that time, applying clinical reasoning principles and relevant evidence when appropriate.”

Following this, ChatGPT received the patient's history and subjective examination findings and was asked the first question:

Subjective (S): *What are your hypotheses and reasoning regarding the dominant pain type (nociceptive, neuropathic, nociplastic), possible sources of symptoms, and specific pathology?*

After ChatGPT responded, the second question was posed:

Objective (O): *Based on your subjective hypotheses, how would you structure your objective examination to confirm or refute your clinical suspicions? Please describe the key components you would include and justify their relevance.*

Next, objective examination findings were provided, followed by the third question:

Assessment (A): *Do the findings of the physical examination* *support your earlier thoughts about the dominant pain type and likely source of symptoms? Please explain how the objective data confirm or challenge your initial hypotheses.*

Finally, the last question was posed:

Plan (P): *Based on the findings from both the subjective and objective examination, and relevant research evidence, what would be your initial management plan? Please justify your intervention choices and short‐term goals.*

This stepwise structure simulated a realistic clinical reasoning process and allowed ChatGPT to respond based on incrementally revealed clinical data. Responses were evaluated by two licensed physical therapists with a clinical doctorate degree (DPT), and consensus ratings were used as the final scores. Accuracy was evaluated using a five‐point Likert scale (1—completely incorrect; 2—more incorrect than correct; 3—approximately equal correct and incorrect; 4—more correct than incorrect; 5—completely correct), and completeness was rated using a three‐point Likert scale (1—incomplete, addresses some aspects of the question, but significant parts are missing or incomplete; 2—adequate, addresses all aspects of the question and provides the minimum amount of information required to be considered complete; 3—comprehensive, addresses all aspects of the question and provides additional information or context beyond what was expected), based on previously established criteria (Rossi et al. 2024).

## 3 Results

Table 1 is a summary of ChatGPT's performance in clinical reasoning across the 10 musculoskeletal cases evaluated using the four components of the SOAP model. Accuracy scores across the four components ranged from 3 to 5 among the 10 cases. Mean accuracy scores were highest in the Assessment (4.4), followed by Subjective (4.3), Plan (4.3), and Objective (3.7). Completeness scores across the four components ranged from 1 to 3 with a similar trend. Mean completeness scores were highest in the Assessment (2.8), followed by Subjective (2.4), Plan (2.4), and Objective (2.0). Overall, this study suggests that while ChatGPT demonstrates strengths in synthesising and interpreting clinical information, particularly in the Assessment component, its performance remains suboptimal and less consistent when applied to more nuanced, context‐specific aspects of clinical reasoning.

**TABLE 1:** ChatGPT's accuracy and completeness scores across ten musculoskeletal cases.

Cases	Subjective		Objective		Assessment		Plan	
	Accuracy	Completeness	Accuracy	Completeness	Accuracy	Completeness	Accuracy	Completeness
Achilles tendinopathy	4	2	4	2	5	3	4	2
Headache	5	2	3	1	4	2	5	3
Hip pain	5	3	4	3	5	3	5	3
Lateral elbow pain	5	3	4	2	5	3	4	3
Lower back pain	3	1	3	1	4	3	4	2
Knee pain	5	3	3	2	4	3	4	1
Neck pain	5	3	4	2	5	3	5	3
Orofacial pain	4	3	3	2	4	3	4	3
Shoulder pain	4	2	5	3	5	3	5	2
Thoracic spine pain	3	2	4	2	3	2	3	2
Mean score	4.3	2.4	3.7	2.0	4.4	2.8	4.3	2.4

## 4 Discussion

ChatGPT's lowest performance in both accuracy and completeness was observed in the Objective domain, suggesting difficulties in formulating patient‐specific examination strategies. Rather than tailoring assessments to individual symptom profiles, ChatGPT often produced generic lists of tests and measures without considering factors such as symptom irritability, acuity, or stage of recovery. This lack of clinical prioritisation may result in examination procedures that risk symptom exacerbation or lack of diagnostic efficiency. The incorporation of clinical reasoning frameworks such as SINSS (severity, irritability, nature, stage, stability) (Petersen et al. 2021) could help structure more appropriate and tolerable assessments. While ChatGPT demonstrated a broad understanding of examination content, it frequently lacked the nuanced sequencing and contextual judgement necessary to guide a safe and targeted physical examination.

Furthermore, ChatGPT did not consistently address red flag screening for spinal conditions, a critical step to ensure patient safety, in the context of direct access physical therapy. Well‐established tools such as the OSPRO (George et al. 2018) and IFMOPT cervical framework (Rushton et al. 2023) should be integrated into subjective and objective examination processes to support effective triage and referral when necessary. Similarly, interactions between symptoms and pharmacologic factors (e.g., medication overuse‐related headache) were not considered, reflecting a gap in integrative reasoning that combines medical history with presenting symptoms.

In the Plan component, responses often lacked appropriate progression strategies and failed to incorporate condition‐specific nuances. For instance, in a case involving knee pain with a dominant nociplastic mechanism, ChatGPT did not recommend initiating strengthening from proximal segments before engaging the symptomatic joint—an approach commonly used to improve load tolerance and reduce symptom exacerbation. Similarly, ChatGPT did not adequately appreciate the pathokinesiologic distinctions between insertional and midportion Achilles tendinopathy, overlooking important implications for exercise prescription, such as the need to initiate exercises on a flat surface in insertional cases (de Vos et al. 2021). These examples suggest that while ChatGPT demonstrates a broad understanding of therapeutic objectives, it struggles to apply stage‐specific management principles and adapt interventions to the biomechanical characteristics of distinct tissue presentations.

These findings underscore important cautions regarding ChatGPT's performance in clinical reasoning. While ChatGPT can simulate structured responses and offer plausible hypotheses, it lacks the depth of contextual judgement required for safe and effective clinical decision‐making. Its omission in triaging red flags, tailoring objective tests and measures, and incorporating nuanced treatment planning highlights the risk of relying on AI‐generated reasoning without expert oversight. The outputs may appear coherent and convincing, yet they may omit essential safety checks or misrepresent best practices. Moreover, ChatGPT's ability to incorporate clinical guidelines and best practices remains questionable, given the variability of its training sources, the potential for outdated information, and the absence of explicit citation or source transparency (Barbosa‐Silva et al. 2025).

As ChatGPT and similar AI tools become increasingly ubiquitous in educational and clinical settings, it is essential for physical therapy educators, both academic faculty and clinical instructors, to emphasise the development of students' critical thinking, evidence retrieval, and appraisal skills as foundational to clinical reasoning. Therefore, at this stage, ChatGPT should not be viewed as an independent clinical tutor or reasoning engine. Instead, its continued development and optimization must involve collaboration with physical therapy clinicians and educators who can provide domain‐specific insight, identify content gaps, and ensure alignment with professional standards and patient safety principles. Without this expert involvement, there is a risk that such AI tools may reinforce incomplete, inconsistent, or oversimplified reasoning patterns that are not appropriate for physical therapy education and clinical training and may ultimately compromise the quality of patient care.

## Author Contributions

**Jie Hao:** conceptualisation, data curation, formal analysis, investigation, methodology. **Zixuan Yao:** conceptualisation, data curation, formal analysis, investigation, methodology. **Ka‐Chun Siu:** conceptualisation, formal analysis, investigation, methodology, supervision.

## Ethics Statement

The authors have nothing to report.

## Conflicts of Interest

The authors declare no conflicts of interest.

## Data Availability

Data sharing not applicable to this article as no datasets were generated or analysed during the current study.
